# Primary resection anastomosis versus Hartmann’s procedure in Hinchey III and IV diverticulitis

**DOI:** 10.1186/s13017-019-0251-4

**Published:** 2019-07-11

**Authors:** Hosam Halim, Alan Askari, Rebecca Nunn, James Hollingshead

**Affiliations:** West Hertfordshire Hospitals NHS Trust, Watford General Hospital, Vicarage Road, Watford, WD18 0BU UK

**Keywords:** Diverticula, Peritonitis, Hartmann’s procedure, Resection and anastomosis

## Abstract

**Introduction:**

Surgical management of Hinchey III and IV diverticulitis utilizes either Hartmann’s procedure (HP) or primary resection anastomosis (PRA) with or without fecal diversion. The aim of this meta-analysis is to determine which of the two procedures has a more favorable outcome.

**Methods:**

A systematic review of the existing literature was performed using the PRISMA guidelines. A meta-analysis was carried out using a Mantel-Haenszel, random effects model, and forest plots were generated. The Newcastle-Ottawa and Jadad scoring tools were used to assess the included studies.

**Results:**

A total of 25 studies involving 3546 patients were included in this study. The overall mortality in the HP group was 10.8% across the observational studies and 9.4% in the randomized controlled trials (RCTs). The mortality rate in the PRA group was lower than that in the HP group, at 8.2% in the observational studies and 4.3% in the RCTs. A comparison of PRA vs HP demonstrated a 40% lower mortality rate in the PRA group than in the HP (OR 0.60, 95% CI 0.38–0.95, *p* = 0.03) when analyzing the observational studies. However, meta-analysis of the three RCTs did not demonstrate any difference in mortality, (OR 0.44 (95% CI 0.14–1.34, *p* = 0.15). Wound infection rates between the two groups were comparable (OR 0.75, 95% CI 0.20–2.78, *p* = 0.67).

**Conclusion:**

Analysis of observational studies suggests that PRA may be associated with a lower overall mortality. There were no differences in wound infection rates. Based on the current evidence, both surgical strategies appear to be acceptable.

## Introduction

Diverticulitis is inflammation of the diverticula of the colon, occurring in approximately 20% of all those who have them [1, 2]. By the sixth decade of life, approximately 40% of the population will develop diverticular pockets [3], accounting for a considerable proportion of healthcare expenditures [4]. Uncomplicated diverticulitis is regarded as colonic inflammation restricted to the bowel wall and mesocolon, in contrast to complicated diverticulitis which results in pericolic, distant intra-abdominal abscesses, or diffuse peritonitis. To classify disease severity, a number of classification systems have been proposed, the most widely used being the Hinchey classification (Table 1).

**Table 1:** Modified Hinchey classification

Modified Hinchey stage	Features
Stage 0	Diverticula with or without wall thickening of the colon
Stage 1	Diverticulitis with a confined pericolic phlegmon or abscess
Stage 1a	Phlegmon with inflammatory reaction in pericolic fat tissue
Stage 1b	Confined pericolic abscess (< 5 cm) close to the inflammatory site
Stage 2	Diverticulitis with abscess distant from the primary inflammatory site (intra-abdominal, retroperitoneal, or pelvic)
Stage 2a	Amenable to percutaneous drainage
Stage 2b	Complex abscess associated with a possible fistula
Stage 3	Generalized purulent peritonitis
Stage 4	Fecal peritonitis

For Hinchey stages I and II, conservative treatment or medical therapy in the form of analgesia and antibiotics are generally recognized as sufficient to control symptoms. For stages 3 and 4, however, there is less agreement on the best treatment modality. After resection of the afflicted colonic segment, the options are to exteriorize the bowel (i.e., Hartmann’s procedure [HP]) or perform an anastomosis (i.e., primary resection anastomosis [PRA]) with or without a covering (de-functioning) loop ileostomy. Colonic lavages in which the bowel is washed out intra-operatively may also be performed when an anastomosis is being created. Over the last few decades, a significant amount of colorectal literature has been dedicated to reporting the outcomes related to all of these options.

Given that patients with Hinchey stages III and IV are often physiologically in extremis and require emergency surgery, the stakes are high, and identifying a treatment modality that would offer the lowest rates of mortality and morbidity would obviously be beneficial. However, no clear advantage has been demonstrated between HP and PRA. The aim of this systematic review and meta-analysis is to determine whether PRA or HP has a better morbidity and mortality profile in the treatment of Hinchey III and IV diverticulitis.

## Method

This systematic review and meta-analysis was carried out in line with the preferred reporting in systematic reviews and meta-analysis (PRISMA) recommendations.

### Search strategy

Two authors independently carried out literature searches using the Embase, PubMed, and Google Scholar datasets. A combination of the following MeSH headings was used: diverticul*, anastomosis, peritonitis, resection, and Hartmann*. Manual searching of references for relevant articles was also performed.

### Inclusion and exclusion criteria

Comparative studies written in the English language that included data on patients who had Hinchey III and IV diverticulitis as reported or described by the authors were included. Case reports, letters, commentary, and abstracts were excluded.

### Quality assessment

All selected studies were quality assessed using the validated Newcastle-Ottawa Scale (NOS) for observational studies and the Jadad Score for Randomized Control Trials (RCTs). Two of the authors, HH and RN, scored the studies independently of each other, and an average of the two scores was taken.

### Statistical analysis

All statistical analyses were carried out using the Cochrane Review Manager (RevMan) version 5.0. Random effect models using the Mantel-Haenszel method were used, and forest plots were generated. The results are reported as odds ratios (ORs) with the accompanying measure of uncertainty as 95% confidence intervals (CIs). A value of *p* ≤ 0.05 was considered statistically significant. The level of heterogeneity is reported and was determined using the chi-square method. Funnel plots were generated to test for potential publication bias.

## Results

**Fig. 1:**
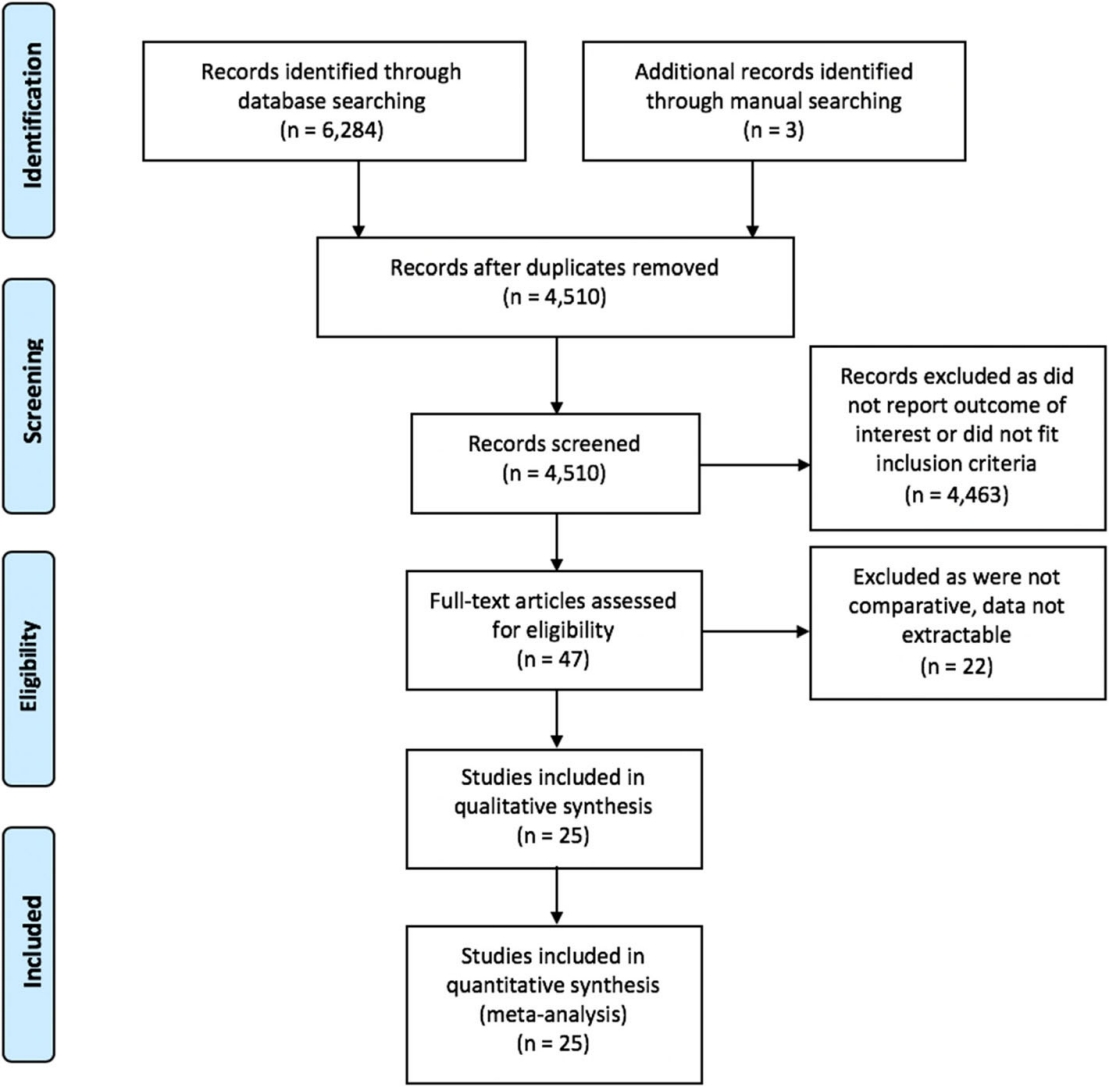
PRISMA flow diagram of the search strategy

Electronic searching using the above terms returned a total of 6284 articles, and an additional three articles were identified through manual searching of abstracts (Fig. 1). Among these 6287 articles, 1774 were duplicates, leaving a total of 4510 abstracts that were screened. Of these, 4463 were excluded because they did not meet the inclusion criteria due to not reporting outcomes of interest or because they, were case reports, techniques, commentary, or letters to the editor leaving a total of 47 articles that were fully examined for eligibility. Among these, an additional 22 articles were excluded because they were either not comparative studies or the relevant data were not presented in a manner allowing for data extraction. The process left a total of 25 studies, of which 22 were observational studies [5–26] and three were (RCTs) [27–29] to be included in this meta-analysis.

The 25 studies included 3546 patients, of whom 2868 underwent HP and 860 underwent PRA. The overall mortality in the HP group was 10.8% across the observational studies and 9.4% in the RCTs. The mortality rates in the PRA group, at 8.2% in observational studies and 4.3% in the RCTs, were lower than those in the HP group (Table 2).

**Table 2:** Study demographics and outcomes for the HP and PRA group

Observational studies															
Belmonte	1996	27	NR	NR	1	3.7%	Open	85	3	NR	NR	16	2	2.4%	Open
Berry	1989	6	NR	NR	3	50.0%	Open	1	NR	NR	NR	NR	0	0.0%	Open
Binda	1993	17	NR	1	6	35.3%	Open	9	NR	1	NR	NR	1	11.1%	Open 30, lap 4
Drumm	1984	5	NR	NR	1	20.0%	Open	3	NR	NR	NR	NR	2	66.7%	NR
Gawlick	2012	1678	NR	226	104	6.2%	NR	340	NR	40	NR	210	19	5.6%	Open
Gooszen	2001	19	NR	NR	4	21.1%	Open	21	NR	NR	NR	32	3	14.3%	Open
Hold	1990	31	NR	NR	6	19.4%	Open	16	NR	NR	NR	5	3	18.8%	Open
Makela	2002	47	NR	NR	6	12.8%	Open	1	NR	NR	NR	NR	0	0.0%	Open
Medina	1991	3	NR	NR	1	33.3%	Open	3	0	0	NR	NR	0	0.0%	Open
Nagorney	1985	84	NR	NR	6	7.1%	Open	4	NR	NR	NR	4	0	0.0%	Open
Peoples	1990	25	NR	NR	6	24.0%	Open	11	0	NR	NR	NR	2	18.2%	Open
Regenet	2003	33	5	9	4	12.1%	Open	27	3	2	3	27	3	11.1%	Open
Richter	2006	5	NR	NR	3	60.0%	Open	36	1	NR	NR	3	4	11.1%	Open
Saccomani	1993	7	NR	NR	3	42.9%	Open	11	0	NR	NR	5	1	9.1%	Open
Schilling	2001	42	3	NR	3	7.1%	Open	13	NR	NR	NR	NR	1	7.7%	Open
Thaler	2000	62	NR	NR	22	35.5%	Open	20	NR	NR	NR	NR	4	20.0%	Open
Trenti	2011	60	NR	19	27	45.0%	Open	27	3	10	NR	5	2	7.4%	Open
Tucci	1996	7	NR	NR	1	14.3%	Open	1	NR	NR	0	NR	0	0.0%	Open
Tudor	1994	40	NR	NR	10	25.0%	Open	8	0	NR	NR	NR	6	75.0%	Open
Vennix	2016	240	NR	NR	18	7.5%	Open	67	NR	NR	NR	NR	0	0.0%	Open
Vermeulen	2007	95	NR	NR	36	37.9%	Open	26	NR	NR	NR	NR	7	26.9%	Open
Wedell	1997	15	NR	NR	4	26.7%	Open	14	0	NR	NR	4	1	7.1%	Open
		2548	8	255	275	10.8%		744	7	53	3	295	61	8.2%	
Randomized control trials (RCTs)															
Binda	2012	56	NR	20	6	11%	Open 53,lap 3	34	1	15	NR	34	1	2.9%	Open
Bridoux	2017	52	NR	NR	3	6%	Open	50	2	NR	NR	50	1	2.0%	Open
Oberkofler	2012	30	NR	NR	4	13%	Open	32	NR	NR	NR	32	3	9.4%	Open
		138	0	20	13	9.4%		116	3	15	0	116	5	4.3%	

### Mortality

**Fig. 2:**
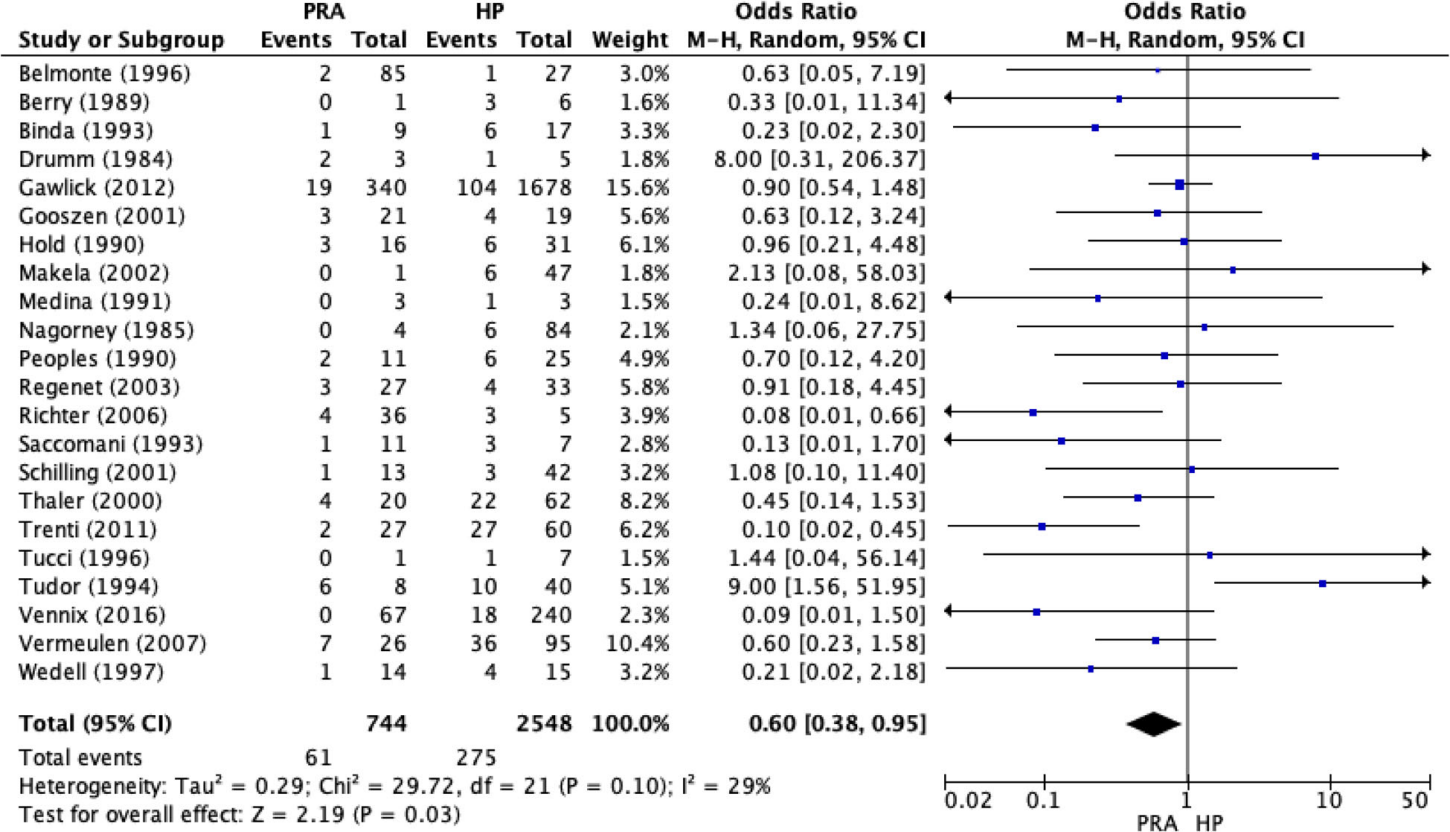
Mortality for the PRA and HP groups as reported in observational studies

**Fig. 3:**
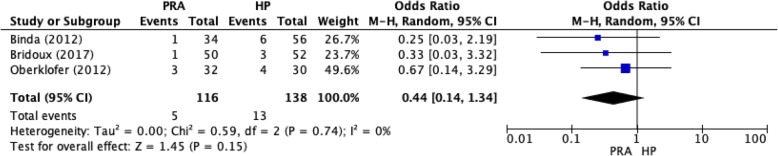
Mortality for the PRA and HP groups as reported in RCTs

Meta-analysis of the 22 observational studies (Fig. 2) demonstrated lower overall mortality in the PRA group than in the HP group (OR 0.60, 95% CI 0.38–0.95, *p* = 0.03). There was a low level of heterogeneity (*I*^*2*^=29, *p* = 0.10). However, the RCTs did not demonstrate a statistically significant difference in mortality between the PRA and HP groups (Fig. 3).

### Wound infections

**Fig. 4:**
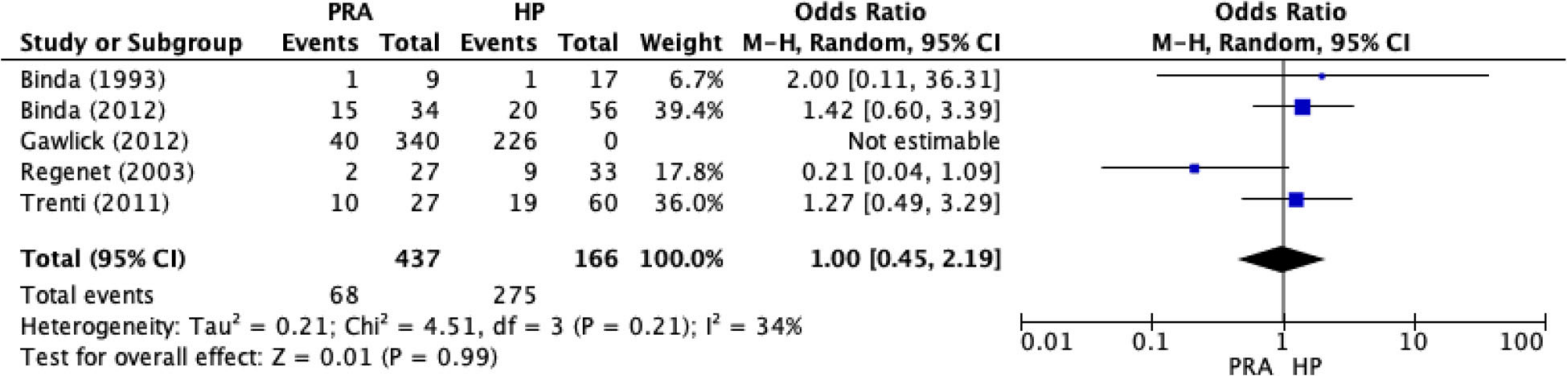
Wound infection rates for the PRA and HP groups

Five studies (all observational) provided data on wound infection rates [13, 17, 21, 27]. A total of 2624 patients (Fig. 4) were included in the analysis, of whom 1844 underwent HP and 437 underwent PRA (Table 3). The rate of wound infection was 15.6% (*n* = 68/437) in the PRA group compared with 14.9% (*n* = 275/1844) in the HP group, although this difference was not statistically significant (*p* = 0.17).

**Table 3:** Wound infection rates between HP and PRA

Author	Year	Hartmann (*n*)	Hartmann’s wound infection	PRA (*n*)	PRAs wound infection
Binda	1993	17	1	9	1
Binda	2012	56	20	34	15
Gawlick	2012	1678	226	340	40
Regenet	2003	33	9	27	2
Trenti	2011	60	19	27	10
		1844	275	437	68
		14.9%		15.6%	

### Stoma complications and anastomotic leakage

Only two observational studies by (Regenet [8] and Schilling [11]) provided data regarding stoma complications. The stoma complication rate across these two studies was 10.7% (*n* = 8/75). In the PRA group, 10 studies [7–10, 13, 15, 17, 19, 25, 28] involving 221 patients provided data suitable for analysis. Of these 221 patients, 10 experienced an anastomotic leak (4.5%). From the data provided, it was not possible to discern how many of these patients had defunctioned loops, how many had clinical or radiological leaks, or how the leaks were treated.

### Study quality

Overall, the studies scored moderately in terms of quality. For the observational studies, only one study scored 8 points out of a maximum of 9 (Makela [24]). Four other studies scored 6 points (Peoples [7], Regenet [8], Trenti [13] and Tucci [14]) , while the rest scored five or four points. No observational study scored less than 4 points. The RCTs scored relatively poorly using the Jadad scale; Oberkofler [29] scored 4 points, while Binda [27] and Bridoux [28] scored 3 points each out of a maximum of 7 points available.

## Discussion

The results of this systematic review and meta-analysis suggest that patients who undergo primary resection with anastomosis may have a lower rate of mortality than patients who undergo HP do; however, this finding is not corroborated by the RCTs included in the meta-analysis. Wound infection rates were not different between the two groups, although this finding is dependent on observational study data only. The findings of this systematic review are similar (in terms of mortality) to previously published studies, almost all of which have reported modest or little difference in terms of mortality between the PRA and HP patient groups [30–37].

There has long been conflicting evidence in the literature as to whether there is a genuine difference in terms of morbidity and mortality between PRA and HP for patients with Hinchey III and IV diverticulitis. Over the past 20 years, several studies have reported that both mortality and morbidity are lower in patients who are treated with PRA [8, 27, 28]. Of the 22 observational studies included in this meta-analysis, only 3 studies reported a higher mortality in the PRA group [11, 15, 20]. The other 19 studies all reported a higher mortality in the HP group. However, these results must be interpreted with some caution. It is possible that an element of case selection or selection bias has influenced the results. Given that patients with Hinchey stages III and IV are usually in a poor physiological state (due to sepsis, dehydration, systemic inflammatory response, etc.), it is entirely possible and arguably even probable that patients selected for PRA were generally more physiologically robust patients and had fewer comorbidities, hence the perceived favorable outcome. Significant case selection bias for PRA would be expected to result in a greater difference in mortality between HP and PRA in the observational studies than in the RCTs. Interestingly, the mortality benefit of PRA over HP was greater in the RCTs than in the comparative studies but was only statistically significant in the analysis of the larger number of patients recruited to the comparative studies.

None of the RCTs conducted have demonstrated that PRA with or without covering stoma is superior to HP in terms of mortality. In the RCT conducted by Binda and colleagues, patients were randomized from 14 centers in 8 countries, and in the 9-year duration of the study, no difference in mortality was observed [27]. Notably, PRA has also not been demonstrated to be inferior either, suggesting that either modality is acceptable in patients with Hinchey stages III and IV.

The main concern in patients undergoing PRA is anastomotic integrity. While there are a number of patient characteristics and disease factors that contribute to anastomotic breakdown, in the context of acute diverticulitis, a combination of bacterial peritonitis and a fecal-loaded colon appear to contribute to anastomotic leakage [38]. Intra-operative colonic lavage has been reported to reduce complications after surgery [8], and there has been some evidence in animal studies that fecal loading can contribute to anastomotic leakage [39, 40]. However, this finding has not been convincingly reproduced in human studies.

The choice between PRA and HP in the presence of perforating diverticulitis depends largely on the severity of inflammation, the intra-operative findings and the surgeon’s comfort with the level of risk. HP is generally believed to be the less risky and safer of the two strategies and tends to be the default option for patients who have profound physiological disturbance/sepsis or are elderly and frail. As a result, patients undergoing HP are often reported as having a higher rate of post-operative infection and mortality [41], and many do not exhibit a reversal of their stoma [42].

Measures to reduce the risk of anastomotic leakage, or at least mitigate the consequences have been used, mainly through the incorporation of diverting ileostomies. Critics of such an approach may state that the whole point of PRA was to avoid a stoma in the first instance, with the argument if there is a need to have a stoma, why not to perform an HP. The counter-argument to this strategy would be that while loop ileostomies still require reversal, they are generally far less technically challenging and do not require entry into the abdominal cavity, which is associated with significant morbidity, including a 14% risk of complications and a 4% risk of anastomotic leakage [42]. Several observational studies have demonstrated that PRA with diverting ileostomy offers a lower mortality risk as well as a higher rate of stoma reversal [17, 29, 43], but again, the likelihood of case selection bias remains a problem.

Mortality and anastomotic leak are not the only conditions one must consider when faced with the difficult task of choosing an operative strategy for Hinchey stages III and IV. Post-operative complications such as respiratory tract infection, urinary tract infection, cardiorespiratory complications, and venous thrombo-embolism remain a problem in this patient group. Some authors have reported lower complication rates and lengths of stay with PRA than with HP [43], although this result is also likely to be influenced by case selection.

As with any systematic review, our study has numerous limitations. The vast majority of the studies (22 out of 25) were observational studies, the quality of which was extremely variable as demonstrated by the quality scoring. It is often difficult to extrapolate from the data presented in these types of studies, specifically the data on the comorbidity status of the populations, the surgical approach used, the extent of peritoneal soiling/intra-operative findings, any other interventions the patient groups may have had or access to higher levels of care such as the intensive care unit or high-dependency unit. All of these factors undoubtedly play a major role in post-operative morbidity and mortality. The populations presented in the studies and the treatments they received were from a variety of different countries and healthcare systems: therefore, they are unlikely to be homogenous. It was also not always clear as what imaging modality (if any) was used by authors to identify patients with Hinchey stages III and IV.

Within the confines of these accepted limitations, PRA with or without diversion ileostomy appears to offer a better mortality rate but no difference in wound infection rates. However, these findings are not corroborated by RCTs, and there may be an element of selection bias that has led to these findings. Based on the current evidence and in the context of Hinchey stages III and IV diverticulitis, both surgical strategies are equally acceptable.

## Conclusion

Analysis of observational studies suggests that PRA may be associated with a lower overall mortality: however, this was not reproduced upon analysis of several RCTs. There were no differences in wound infection rates. There may be an element of case selection bias in comparative observational studies, but the low mortality in the PRA group across the RCTs suggests this difference is not significant. Based on the current evidence, both surgical strategies appear to be acceptable.

## Data Availability

Not applicable.

## Abbreviations

CI: Confidence interval
HP: Hartmann’s procedure
NOS: Newcastle-Ottawa scale
OR: Odds ratios
PRA: Primary resection anastomosis
PRISMA: Preferred reporting in systematic reviews and meta-analysis
RCTs: Randomized control trials
RevMan: Review Manager
